# 465. Mental Health Treatment Disparities During the COVID-19 Pandemic

**DOI:** 10.1093/ofid/ofab466.664

**Published:** 2021-12-04

**Authors:** Katherine Kricorian, Karin Kricorian

**Affiliations:** MiOra, Los Angeles, CA, Simi Valley, California

## Abstract

**Background:**

The COVID-19 pandemic has been associated with a decline in mental health status in the US, as well as reduced ability to seek mental health treatment. This study analyzed undertreatment of mental health during the pandemic to identify possible disparities and assess the need for interventions.

**Methods:**

Data were collected from Wave 3 (January 6-February 15, 2021) of the US Census COVID-19 Household Pulse online survey, designed to measure the ongoing impact of the pandemic. Microdata files were downloaded from the Census website and included N=185,201 respondents. Data was collected in both English and Spanish and consisted of a representative sample of US residents. Data were analyzed using χ ^2^ tests, with z-tests for more granular between-group comparisons.

**Results:**

When asked if they needed and received therapy due to mental health concerns, 81% of respondents did not need therapy and did not receive it. Some (2%) reported receiving therapy but needing more. However, 9% reported needing therapy but not receiving it. A similar proportion, 9%, reported having received adequate therapy from a mental health professional. Those who needed therapy but did not receive it were more likely than adequately treated respondents to express debilitating worry, anxiety, depression, and lack of interest/pleasure in doing things (all p< .05). These respondents were also more likely (vs. adequately treated respondents) to be younger, lower-income, racial/ethnic minorities, without health insurance, and food-insecure (all p< .05).

**Conclusion:**

Inadequate mental health treatment is a critical challenge, especially in the wake of COVID-19; Just as many respondents reported adequate mental health treatment as did needing additional mental health treatment. Respondents reporting undertreated mental health issues in this study were more likely to be vulnerable populations, many of whom have already been disproportionately impacted by the pandemic. Methods to expand accessible counseling capacity in economically feasible ways to limit these disparities should be further explored.

**Disclosures:**

**All Authors**: No reported disclosures

